# Obstetric risk indicators for labour dystocia in nulliparous women: A multi-centre cohort study

**DOI:** 10.1186/1471-2393-8-45

**Published:** 2008-10-06

**Authors:** Hanne Kjærgaard, Jørn Olsen, Bent Ottesen, Per Nyberg, Anna-Karin Dykes

**Affiliations:** 1 Juliane Marie Centre for Women, Children and Reproduction, Copenhagen University Hospital, Rigshospitalet, Denmark; 2 Department of Epidemiology, School of Public Health, University of California, Los Angeles, USA; 3 Department of Health Sciences, Faculty of Medicine, Lund University, Sweden

## Abstract

**Background:**

In nulliparous women dystocia is the most common obstetric problem and its etiology is largely unknown. The frequency of augmentation and cesarean delivery related to dystocia is high although it is not clear if a slow progress justifies the interventions. Studies of risk factors for dystocia often do not provide diagnostic criteria for the diagnosis. The aim of the present study was to identify obstetric and clinical risk indicators of dystocia defined by strict and explicit criteria.

**Methods:**

A multi-centre population based cohort study with prospectively collected data from 2810 nulliparous women in term spontaneous labour with a singleton infant in cephalic presentation. Data were collected by self-administered questionnaires and clinical data-records. Logistic regression analyses were used to estimate adjusted Odds Ratios (OR) and 95% confidence intervals (CI) are given.

**Results:**

The following characteristics, present at admission to hospital, were associated with dystocia during labour (OR, 95% CI): dilatation of cervix < 4 cm (1.63, 1.38–1.92), tense cervix (1.31, 1.04–1.65), thick lower segment (1.32, 1.09–1.61), fetal head above the inter-spinal diameter (2.29, 1.80–2.92) and poor fetal head-to-cervix contact (1.83, 1.31–2.56). The use of epidural analgesia (5.65, 4.33–7.38) was also associated with dystocia.

**Conclusion:**

Vaginal examinations at admission provide useful information on risk indicators for dystocia. The strongest risk indicator was use of epidural analgesia and if part of that is causal, it is of concern.

## Background

It remains difficult to determine whether a period of slow progress in labour is pathological and therefore justifies treatment, or is a normal variation in the physiological process leading to delivery, especially when the fetal head is above the inter-spinal diameter and the fetal head-to-cervix contact is poor.

Most interventions during nulliparous labour use dystocia as indication and about 50% of all cesarean deliveries are related to dystocia [1,2]. The term dystocia is used by some authors exclusively when immediate instrumental or cesarean delivery is indicated [1], while others, including ourselves, use the term when augmentation is needed regardless of subsequent mode of delivery [3-6]. The incidence of dystocia is not well monitored and there is no consensus on the length of normal labour or the diagnostic criteria for dystocia [1,3,4,7-11]. An increase in the need for augmentation has been reported in affluent countries and some studies show augmentation is now used in around 50% of nulliparas [6,12-14].

The reasons for the increased incidence of dystocia are only partly known. Poor head-to-cervix force may be associated with slow progress of labour [15,16], as may poor engagement of fetal head at onset of labour [17]. High fetal weight may increase the risk of dystocia [18-20], and it is debated whether epidural analgesia in itself prolongs labour [18,21-27]. With the increasing age in nulliparous women, sub-fecundity is also more frequent and elevated risk of failure to progress was found in fertility-treated pregnancies [18,28]. Iatrogenic factors have also been used to explain the increase in dystocia incidence, augmentation and cesarean delivery [29-32]. Most reported associations between dystocia and obstetric risk factors are based on varying criteria for dystocia; often, no criteria are given. We therefore conducted this study, based on strict and explicit criteria, to identify obstetric and clinical risk indicators for dystocia.

## Methods

Data stem from the Danish Dystocia Study, a population based multi-centre study on incidence, risk indicators and women's experiences of labour with dystocia. Participants were part of a fixed cohort of nulliparous women followed from gestational week 37 through 2 weeks after delivery [33]. The final study population comprised 2810 nulliparous women who delivered a singleton infant in cephalic presentation at term after spontaneous onset of labour. The study was restricted to these nulliparas to reduce co-morbidity that could justify induction or a planned cesarean delivery.

Inclusion into the study took place between May 2004 and July 2005. Participants were recruited from four major university hospitals, three county hospitals and two local district departments with annual birth rates varying between 850 and 5400. Recruitment took place in the antenatal clinics at 33 gestational weeks and baseline information was collected at 37 gestational weeks. In order to have a well defined group with no obvious risk factors for dystocia, the following inclusion criteria were used: nulliparas, 18 years of age or older, Danish speaking, singleton pregnancy, no planned elective cesarean delivery or induction of labour. From inclusion at gestational week 33 to collection of baseline data in gestational week 37, 202 women were excluded for the following reasons: preterm delivery (n = 176), incorrect inclusion (n = 8), multiple pregnancy (n = 9), planned elective cesarean delivery or induction (n = 9).

**Figure 1:**
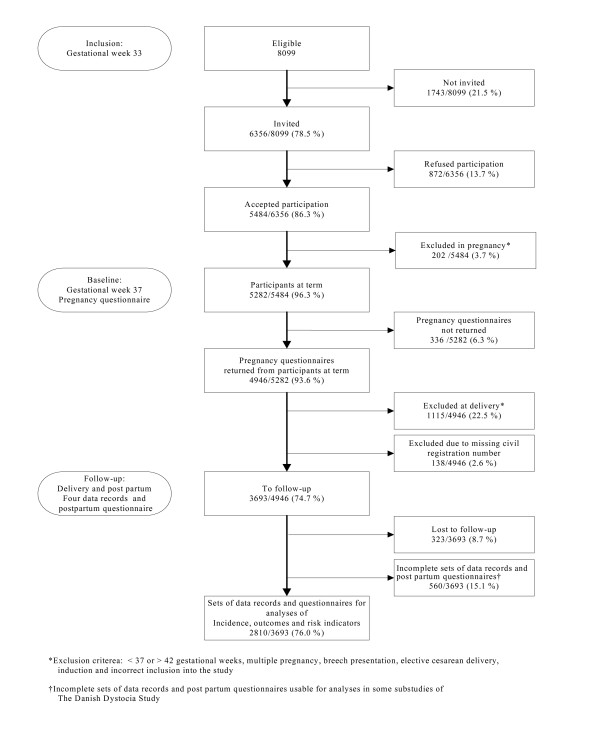
**The Danish Dystocia Study Flowchart**.

Exclusion criteria at delivery were: > 42+0 weeks of gestation, induction, elective cesarean delivery and breech presentation (Figure 1). In total 1115 were excluded at delivery. The reasons for exclusion were as follows: induction (including post-term pregnancies) (n = 741), elective caesarean delivery (n = 84), breech presentation (n = 178) and miscellaneous (n = 112). The latter comprised incorrect inclusion (e.g. non-Danish speaking, < 18 years at inclusion), foetus mortuus and unspecified. In addition we excluded 138 with no civil registration number, as we would not be able to extract data from the Danish National Birth Register to validate their information. Incomplete sets of data (n = 560) were not included in the analyses and 323 were lost to follow up due to an excessive workload for the midwives (n = 274) and miscellaneous (moved or referred to a hospital outside the participating hospitals, declining further participation and unspecified, n = 49).

Diagnostic criteria for dystocia are presented in Table 1. These criteria are in accordance with guidelines from Danish Society of Obstetrics and Gynecology [34], supplemented with a criterion for the descending phase of labour's second stage from the guidelines on dystocia from the American College of Obstetrics and Gynecology [1]. To minimize potential misclassification of the dystocia diagnosis in our study, we performed systematic data quality control measures to assess compliance with the protocol criteria. Dystocia was only given as a diagnosis if the duration of labour exceeded the cut-off times of the criteria in Table 1. Women who received augmentation without fulfilling the study criteria for dystocia (n = 299) were retained in the population at risk. The dystocia diagnosis was not given to women with absence of contractions after prelabour rupture of membranes (PROM) according to the coding guideline of obstetric interventions in Denmark since treatment of PROM is classified as induction of labour [35].

**Table 1:** Definitions of Stages and Phases of Labour and Diagnostic Criteria for Dystocia

**Stage of labour**	**Definition of stage and phase**	**Diagnostic Criteria for Dystocia**
***First stage***	From onset of regular contractions leading to cervical dilatation to full dilatation of cervix	
Latent phase	Cervix 0 – 3.9 cm dilatation	The diagnosis was not to be given in this phase
Active phase	Cervix ≥ 4 cm dilatation	< 1/2 cm dilatation of cervix per hour, assessed over 4 hours = dystocia
***Second stage***	From full dilatation of cervix to the child is born	
Descending phase	From full dilatation of cervix to strong and irresistible urge to push	> 2 hours without descent = dystocia. If epidural is administered: > 3 hours = dystocia
Expulsive phase	Strong and irresistible pushing during the major part of the contraction	> 1 hour without progress = dystocia

Exposure data were collected prospectively. Data collection was based on a self-administered questionnaire completed in gestational week 37. Data records were completed by the assisting obstetric staff at the woman's admission to the labour ward, and during and after labour. Local contact persons at the participating centres undertook close supervision during follow-up in all phases of the data collection.

The examined risk indicators of dystocia were: dilatation and consistency of cervix, thickness of lower segment, descent of fetal head and fetal head-to-cervix contact at admission to the delivery ward, infertility prior to current pregnancy and use of epidural analgesia. Measurements of lower uterine segment, cervix and fetal head conditions were performed manually during routine vaginal examinations. In the pilot phase of the study two methods of validation of vaginal examinations were considered: use of a model or an additional examination by a second midwife. Both methods were discarded as constituting an unacceptable extra workload and also, in the case of the latter, for ethical reasons. We assumed that the weight of the expected child plays a role in the progress of labour. However, the actual weight of the expected child is unknown and cannot with accuracy be taken into consideration for potential clinical management of the labour. Our analyses included as well as excluded, respectively, birth weight in the regression model to examine the effect of birth weight on the clinical risk indicators.

Prior to analyses, dilatation of cervix and birth weight were categorized according to predefined categories. If epidural analgesia was applied after dystocia was diagnosed and augmentation was initiated (n = 47), women were excluded from the risk analyses. The categories of risk indicators are described in Table 2.

**Table 2:** Odds Ratios for dystocia with 95% confidence intervals according to obstetric characteristics

	N 2810	Un-adjusted OR	Adjusted OR* (95% CI)	All variables included	All variables included except birth weight	Trend† OR (95% CI)
Infertility treatment prior to current pregnancy						
No	2449	1 (ref.)	1 (ref.)	1 (ref.)	1 (ref.)	
Yes	184	1.13	0.95 (0.69–1.31)	0.92 (0.65–1.29)	1.09 (0.78–1.53)	
Missing	177					
Dilatation of cervix at admission						0.83 (0.80–0.86)
0–3 cm	1086	1.67	1.63 (1.38–1.92)	1.29 (1.06–1.57)	1.21 (1.00–1.47)	
4–10 cm	1575	1 (ref.)	1 (ref.)	1 (ref.)	1 (ref.)	
Missing	149					
Consistency of cervix at admission						
Tense	585	1.25	1.31 (1.04–1.65)	1.0 (0.79–1.26)	0.98 (0.79–1.23)	
Soft	1794	1 (ref.)	1 (ref.)	1 (ref.)	1 (ref.)	
Missing	431					
Thickness of lower segment at admission						
Thick	583	1.30	1.32 (1.09–1.61)	0.88 (0.69–1.12)	0.91 (0.72–1.14)	
Thin	1712	1 (ref.)	1 (ref.)	1 (ref.)	1 (ref.)	
Missing	515					
Descent of fetal head at admission						
Above the inter-spinal-line	2367	2.33	2.29 (1.80–2.92)	1.80(1.32–2.45)	1.92 (1.42–2.58)	
At or under the inter-spinal-line	311	1 (ref.)	1 (ref.)	1 (ref.)	1 (ref.)	
Missing	132					
Fetal head-to-cervix contact						
Good	1921	1 (ref.)	1 (ref.)	1 (ref.)	1 (ref.)	
Poor	159	1.88	1.83 (1.31–2.56)	1.62 (1.09–2.40)	1.57 (1.08–2.27)	
Cannot be assessed	455					
Missing	275					
Birth weight						1.001 (1.00–1.00)
2000–2499 gr	23	0.14	0.14 (0.32–0.60)	0.27 (0.61–1.21)		
2500–2999 gr	282	0.57	0.51 (0.38–0.69)	0.55 (0.40–0.77)		
3000–3499 gr	1040	0.78	0.74 (0.62–0.89)	0.76 (0.62–0.93)		
3500–3999 gr	1016	1 (ref.)	1 (ref.)	1 (ref.)		
4000–4499 gr	392	1.23	1.29 (1.02–1.65)	1.06 (0.83–1.41)		
≥ 4500 gr	53	1.36	1.37 (0.78–2.41)	1.32 (0.70–2.46)		
Epidural analgesia						
No epidural	2284	1 (ref.)	1 (ref.)	1 (ref.)	1 (ref.)	
Epidural analgesia	316	5.49	5.65 (4.33–7.38)	4.65 (3.53–6.13)	4.77 (3.65–6.22)	

* Crude Odds Ratios controlled for age, height, pre-pregnancy BMI and physical activity, including a variable for the participating departments (9 levels).

† Test for trend performed on continuous variables. First line: Regression coefficient for change in OR per unit increased, second line: 95% CI.

### Ethics

Since no invasive procedures were applied in the study, no Ethics Committee System approval was required by Danish law. The policy of the Helsinki Declaration was followed throughout the data collection and analyses. Written consent was obtained and person-specific data were protected by codes. Permission to establish the database was obtained from the Danish Data Protection Agency j. no. 2004-41-3995.

### Statistical methods

Binary logistic regression analyses were used to estimate crude and adjusted odds ratio (OR) for dystocia with 95% confidence intervals (CI). Adjustments were made for age in four groups (< 25, 25–29, 30–34, 35+), height in three groups (< 160, 160–169, 170+), pre-pregnancy BMI in five groups (< 18.5, 18.5–24.9, 25.0–29.9, 30+) and level of physical activity in first trimester in four groups (regular intensive physical training and competitive sports several times/week, athletics or heavy physical activity ≥ 4 hours/week, light physical activity ≥ 4 hours/week, predominantly sedentary lifestyle) as these potential confounders may be independent risk factors for dystocia, and some are known to interact with the risk factors under study, i.e. BMI and birth weight and age and infertility. Age, height, pre-pregnancy BMI and level of physical activity were initially all included in the model and subsequently deleted one by one with replacement. None of the variables changed the estimate by more than 10% but we decided to keep all variables in the model since they did not increase the variance of the OR. We also estimated OR for the clinical risk indicators with all variables included in the model to adjust for their mutual associations and to identify which factors had the strongest independent association. We calculated trend values for continuous variables (cervix dilatation and birth weight) by using logistic regression. Odds ratio for trend represent a change in OR per unit increase or decrease in the exposures under study. In order to take into consideration potential clinical and social characteristics of the nine participating centres, we adjusted all analyses by including study centre in the model as a dummy variable. Descriptive statistics for continuous variables are presented as means with 95% CI or medians (Inter-quartile range (IQR)) depending on distributional characteristics.

Statistical analyses were performed using SPSS 14.0 software (SPSS Inc., Chicago, Il).

## Results

The mean maternal age was 28.7 years (95% CI; 28.5 – 28.8) at entry into the cohort, but women with dystocia were slightly older (29.1 yrs.). Median pre-pregnancy BMI was 22.3 (IQR 4.2) with no significant difference between the two groups. In this population 84% were non-smokers and 66% were engaged in light physical activity > 4 hours per week.

Table 2 presents the unadjusted and adjusted OR within categories of obstetric risk indicators. Among the risk indicators recorded at the woman's admission to the delivery ward, cervix dilatation < 4 cm, descent of fetal head above the inter-spinal level and poor fetal head-to-cervix contact had the strongest association with dystocia.

Epidural analgesia was also strongly associated with dystocia. The variables related to cervix are closely correlated (part of the same manifestation) and when we included all variables from Table 2 in the statistical model, most estimates were attenuated as expected. After mutual adjustments the estimates that remained strongest were epidural analgesia, descent of fetal head above the inter-spinal diameter, poor fetal head-to-cervix contact and dilatation of cervix < 4 cm at admission.

Expecting a child with a birth weight < 3500 gr. appeared to be protective for dystocia while expecting a child with a birth weight > 4000 gr. was associated with increased risk of dystocia compared to birth weights 3500–3999 gr. The last column presents OR without birth weight included in the statistical model. Birth weight appeared to have a minor effect on OR and confidence intervals of the clinical risk indicators.

## Discussion

The vaginal examinations at the woman's admission to the labour ward provided several prognostic indicators of dystocia later in labour. Expecting a child with a high birth weight and the use of epidural analgesia were also associated with the risk of dystocia, the latter association being particularly strong.

Fetal head above the inter-spinal diameter had the strongest association with dystocia among the factors present at admission of women in labour, also in the analyses that excluded birth weight. Others have found that lack of descent of fetal head often leads to cesarean delivery for dystocia [17,36]. Descent of fetal head is correlated to dilatation of the cervix, and cervix dilatation < 4 cm at admission was associated with an increased risk of dystocia. Women admitted with little cervical dilatation may have unbearably painful contractions. Anxiety may play a role for early admission as well as concern for high maternal blood pressure, fetal heart rates or other clinical conditions. High risk of dystocia in women admitted in early labour has also been found other studies [29,30,37,38], perhaps because early admission introduces risk of iatrogene-induced treatments [29,30]. We believe, however, that quality control of our data prior to analyses ensured that the dystocia diagnosis was registered exclusively in women who met the criteria. We therefore assume that iatrogenic factors have not played a major role in our findings.

A poor fetal head-to-cervix contact at admission was associated with near doubling of the odds of dystocia. Gough et al. found that low head-to-cervix force was associated with poor progress and delivery by cesarean section for dystocia [39]. Allman et al. examined the head-to-cervix force electronically and advocate that head-to-cervix force is a better predictor of the likely rate of cervical dilatation than intrauterine pressure and also a better predictor of mode of delivery than the dilatation rate itself [15,16]. Our findings based on manual assessments during vaginal examinations support Allman's findings of an association between poor head-to-cervix contact and dystocia.

Epidural analgesia had the strongest association with dystocia among the risk indicators assessed. In total 71.2% of women who were treated with epidural analgesia were diagnosed with dystocia. A similarly strong association between dystocia and epidural analgesia was reported from a population-based study of 106,755 deliveries without induction and with durations of delivery < 12 hours [14], but the literature is inconsistent on the effects of epidural analgesia on the course of labour and delivery and maternal and fetal outcomes [18,21-27]. Lower plasma oxytocin levels are found in women with epidural analgesia [40] and this may slow the progress of labour. Alehagen et al. found that women who received epidural analgesia had experienced more fear, but not more pain, before the administration of epidural analgesia than did women who did not receive epidural analgesia [41] and fear may prolong duration of labour [42]. Recent reviews come to the conclusion that epidural analgesia appears to prolong labour's second stage and prompt more use of oxytocin [25-27]. Although we excluded from the analyses those who were treated with epidural analgesia after being diagnosed with dystocia, reverse causation is still a possible explanation of the association we find. If a need for pain relief or fear of pain are among the clinical precursors of dystocia, epidural analgesia could be part of the mechanism leading to dystocia.

We were not able to replicate findings of an association between dystocia and infertility treatment prior to the current pregnancy, perhaps because our statistical power to detect such an association is low. Others have demonstrated a near doubling of the risk of failure to progress in treated women [18].

Our findings of an association between high birth weight (4000–4499 gr.) and dystocia corroborate findings from other studies even though the definition of 'high birth weight' varies [19,20,22]. Although the birth weight can only be estimated before the child is born, the clinical implication of our findings could be that increased risk of dystocia should be considered when an estimated birth weight is more than 4000 gr.

Our findings of association between the clinical conditions dilatation of cervix, descent of fetal head and fetal head-to-cervix contact and dystocia may have significant clinical implications. Women admitted to hospital for delivery have a vaginal examination upon admittance and information from this examination provides the clinical basis for the primary management of labour. Weight of the child appears to have only minor effect on the OR related to the clinical conditions.

### Strengths and limitations

The study has limitations. We reached 78.5% of the women eligible for inclusion in the study and of these 86.3% accepted the invitation to participate. Missed inclusions were mainly a problem during the first months of data collection and we have no reason to believe that this led to over-sampling of women with low or high risk of dystocia as inclusion took place 6–8 weeks before delivery. Almost nine percent were lost to follow up, possibly related to the extra work required from the participating departments. The aims of the Danish Dystocia Study were descriptive as well as analytical and the data collection instruments comprised four detailed data records to be filled in by the obstetric staff during the woman's stay at the delivery ward. It may be that the mere burden of work gave rise to some non-responses to items or non-completion of entire data records. However, we have no reason to assume that non-responses were directly related to risk of dystocia.

We did not include an independent criterion for dystocia based on descent of the fetal head and cases with a quick descent but a slow dilatation may have been classified as dystocia. We recommend that future studies make it possible to identify this group. Evaluation of cervical conditions and descent of fetal head is difficult and subject to considerable intra- and inter observer variation. We recommend that further studies include different methods of measuring conditions related to the cervix and the fetal head (i.e. electronic monitoring).

The study also has important strengths. The population based cohort design, based upon primary and prospectively collected data, is a strength. The risk of differential misclassification was reduced as we used prospectively collected data on cervix and fetal head conditions and the study was based on strict diagnostic criteria agreed upon by all. Central as well as local supervisors took part in all phases of the data collection.

## Conclusion

Our study contributes further evidence of an increased risk of dystocia in nulliparous women who, at admission to hospital, present with a descent of fetal head above the inter-spinal diameter and a cervix dilatation < 4 cm. We found that a tense cervix, a thick lower segment and a poor contact between the fetal head and the cervix are risk indicators for dystocia. Further studies should examine if fetal head-to-cervix contact is a significant predictor of dystocia and if differentiation of the management of dystocia can be based on assessment of fetal head-to-cervix contact. The observed association between epidural analgesia and increased risk of dystocia is of interest and may have a causal explanation.

## Competing interests

The authors declare that they have no competing interests.

## Authors' contributions

HK and AKD planned the study. HK carried out the data collection. Analyses were planned by HK, JO and PN. PN conducted the data validation. HK conducted the analyses. HK, BO, AKD and JO interpreted the results. HK wrote the drafts of the manuscript, which the other authors commented on. All authors approved the final manuscript.

## Pre-publication history

The pre-publication history for this paper can be accessed here:


